# Reply to Guo’s commentary on: “Immune cells lacking Y chromosome show dysregulation of autosomal gene expression”

**DOI:** 10.1007/s00018-021-04028-w

**Published:** 2021-12-15

**Authors:** Lars A. Forsberg

**Affiliations:** 1 Department of Immunology, Genetics and Pathology and Science for Life Laboratory, Uppsala University, Uppsala, Sweden; 2 The Beijer Laboratory, Uppsala University, Uppsala, Sweden

In a commentary on “*Immune cells lacking Y chromosome show dysregulation of autosomal gene expression*” [1], Guo provides insightful comments with focus on the role of mosaic loss of chromosome Y (LOY) in Alzheimer’s disease (AD). The commentary highlights an emerging view that certain somatic genetic variants detected in blood samples could be associated with disease vulnerability in other organs, including risk for AD in men with LOY in leukocytes [2]. Here, three aspects of recent findings discussed in Gou’s commentary are emphasized.

First, an increased risk for disease in men carrying leukocytes without a Y chromosome could be linked with several mechanisms. For example, LOY might directly exacerbate disease progression by impairing normal functions of affected leukocytes. Indeed, LOY-associated transcriptional effects (LATE) have been described on the RNA [1] as well as the protein level [3]. Furthermore, an overlap between germline risk variants for LOY and cancer susceptibility and other conditions suggests that LOY in blood could be viewed as a barometer of genomic imbalance in somatic tissues overall [4]. The relative contribution from direct physiological effects and from a ‘common soil’ of predisposition, respectively, is currently evaluated and likely varies in the etiology of different types of LOY-associated disease. In the commentary, Guo highlights the interesting observation that germline risk variants for LOY and AD appear to be largely non-overlapping. Thus, shared genetic predisposition would not likely explain an increased risk for AD among men with LOY in leukocytes.

Furthermore, based on our results that patients with AD and prostate cancer were primarily affected with LOY in natural killer (NK) cells and CD4 + T lymphocytes and granulocytes, respectively [1], Guo suggests that further analysis of NK cells in AD patients and controls will be informative. It should be noted, however, that although these classes of immune cells showed the highest levels of LOY in our analysis, LOY in other types of immune cells might also be relevant for disease vulnerability. To gain further insight into the phenotypical consequences of LOY and associated disease risks, additional functional studies within all relevant subsets of leukocytes will be useful.

In the commentary, Gou also outlines the possible role of LOY within the infection origin theory of AD. Briefly, it is hypothesized that infectious agents would enter the brain at a higher rate among men affected with LOY in peripheral leukocytes, as a consequence of impaired immune functions. In response to this, brain cells would increase production of peptides such as Aβ to fight infections. Hence, aggregation of Aβ and associated chronic inflammation is a hallmark of AD etiology. If proven accurate by studies designed to test this hypothesis, new preventive measures and treatment options could be envisioned to mitigate this detrimental disease.

## Data Availability

Not applicable.
